# CHALLENGES AND CONSIDERATIONS IN RESEARCH WITH OLDER ADULTS EXPERIENCING HOMELESSNESS

**DOI:** 10.1093/geroni/igad104.3216

**Published:** 2023-12-21

**Authors:** Natalie Pope, Abigail Latimer, Debra Moser, Olivia Sasdi, Hilda Okeyo

**Affiliations:** University of Kentucky, Lexington, Kentucky, United States; University of Kentucky, Lexington, Kentucky, United States; University of Kentucky, Lexington, Kentucky, United States; University of Kentucky, Lexington, Kentucky, United States; University of Kentucky, Lexington, Kentucky, United States

## Abstract

Individuals in their late 50s and 60s constitute the fastest growing cohort of homeless adults; homelessness among the older adult population is expected to triple by 2030 (Culhane et al., 2019). Older adults find themselves homeless due to systemic (e.g., unaffordable housing, structural racism, increasing healthcare costs) and individual factors (e.g., substance misuse, mental health diagnoses). Unhoused older adults have high rates of multiple chronic conditions (Henwood et al., 2018) worsened by unmet healthcare needs (Baggett et al., 2010). Older adults, especially those with serious illness, struggle to find and maintain housing compared to younger adults without illness. Patient and clinician-informed services/programs are needed, but the nature of serious illness and homelessness research comes with unique considerations. Research challenges in this population include inclusive and adequate recruitment, participant screening, and boundary issues (Hanson et al., 2014; Lashley, 2023). We will present lessons learned from completing two successful qualitative studies with unhoused older adults and clinical subject groups. Issues encountered during our research include dealing with recruitment obstacles, evaluating and responding to participants’ cognitive impairment and mental health issues, managing emotional labor experienced by the research team, developing stakeholder relationships, and making decisions about adequate and appropriate study compensation. We describe challenges, opportunities, and suggestions for collecting primary data from unhoused older adults. We encourage gerontological researchers not to avoid this hard-to-reach, high-risk population. The lessons learned in designing and implementing studies tailored to the unique challenges of unhoused older adults can promote inclusion of the perspectives and needs this population.

